# Addressing patient’s unmet social needs: disparities in access to social services in the United States from 1990 to 2014, a national times series study

**DOI:** 10.1186/s12913-022-07749-1

**Published:** 2022-03-19

**Authors:** Yoosun Park, James W. Quinn, Philip M. Hurvitz, Jana A. Hirsch, Jeff Goldsmith, Kathryn M. Neckerman, Gina S. Lovasi, Andrew G. Rundle

**Affiliations:** 1 School of Social Policy and Practice, University of Pennsylvania, Philadelphia, Pennsylvania USA; 2 Department of Epidemiology, Mailman School of Public Health, Columbia University, 722 west 168th street, room 727, New York, New York 10032 USA; 3 Center for Studies in Demography and Ecology, University of Washington, Seattle, Washington USA; 4 Dornsife School of Public Health, Urban Health Collaborative, Drexel University, Philadelphia, Pennsylvania USA; 5 Department of Biostatistics, Mailman School of Public Health, Columbia University, New York, New York USA; 6 Columbia Population Research Center, Columbia School of Social Work, Columbia University, New York, New York USA

**Keywords:** Social needs, Social services, Disparities, Unmet social needs

## Abstract

**Background:**

To address patient’s unmet social needs and improve health outcomes, health systems have developed programs to refer patients in need to social service agencies. However, the capacity to respond to patient referrals varies tremendously across communities. This study assesses the emergence of disparities in spatial access to social services from 1990 to 2014.

**Methods:**

Social service providers in the lower 48 continental U.S. states were identified annually from 1990 to 2014 from the National Establishment Times Series (NETS) database. The addresses of providers were linked in each year to 2010 US Census tract geometries. Time series analyses of annual counts of services per Km^2^ were conducted using Generalized Estimating Equations with tracts stratified into tertiles of 1990 population density, quartiles of 1990 poverty rate and quartiles of 1990 to 2010 change in median household income.

**Results:**

Throughout the period, social service agencies/Km^2^ increased across tracts. For high population density tracts, in the top quartile of 1990 poverty rate, compared to tracts that experienced the steepest declines in median household income from 1990 to 2010, tracts that experienced the largest increases in income had more services (+ 1.53/Km^2^, 95% CI 1.23, 1.83) in 1990 and also experienced the steepest increases in services from 1990 to 2010: a 0.09 services/Km^2^/year greater increase (95% CI 0.07, 0.11). Similar results were observed for high poverty tracts in the middle third of population density, but not in tracts in the lowest third of population density, where there were very few providers.

**Conclusion:**

From 1990 to 2014 a spatial mismatch emerged between the availability of social services and the expected need for social services as the population characteristics of neighborhoods changed. High poverty tracts that experienced further economic decline from 1990 to 2010, began the period with the lowest access to services and experienced the smallest increases in access to services. Access was highest and grew the fastest in high poverty tracts that experienced the largest increases in median household income. We theorize that agglomeration benefits and the marketization of welfare may explain the emergence of this spatial mismatch.

## Introduction

Unmet social needs among patients are associated with greater difficulty in managing chronic diseases, with foregoing medical care, and with higher health care costs [1–5]. Hospitals are increasingly attempting to address patient’s social and economic needs, and in turn improve patient health outcomes, by making referrals to social service agencies and community philanthropic organizations [1, 3, 6–11]. The Centers for Medicaid and Medicare Service’s Accountable Health Communities Model focuses on addressing patient’s health-related social needs through screening and referral of patients to community social service providers [6]. However, the availability of social service agencies and the capacity to respond to these patient referrals varies tremendously across communities [1, 12–15]. It is well established that geographical proximity to social services is crucial to the utilization of those services [15–18]. Prior research has found that nonprofit organizations “tend to locate in somewhat more advantaged areas for a variety of reasons” [19], while the need for their services may be greater in less advantaged areas [14, 20–22]. Notably, Allard and colleagues [15, 17, 23, 24] have called attention to increasing poverty in inner-ring suburbs, where the supply of social services is relatively limited.

Two factors, agglomerative economics and the marketization of welfare, may help explain why social services have been slow to relocate in response to population shifts through time. Agglomeration economics posits that there are advantages when similar businesses and institutions locate near one another and near to other physical and commercial resources that will support the mission of these institutions [25]. Agglomerative effects may create centers of gravity that slow the migration of service providers to new areas. The marketization of welfare describes social service providers’ increasing budgetary reliance on fee for service activities [26]. Service providers who partially rely on fee for service activities may be particularly attracted to high poverty areas that are on an upwards economic trajectory and gaining residents who can afford to pay for services.

Given current strategies to address patient’s social determinants of health through referrals to social service agencies, it is important to understand how spatial access to these services has evolved through time and to identify the processes that shape access. With few exceptions, such as David Clifford’s study of charitable organizations in England from 1996–2011, the extant research on spatial access to social services is overwhelmingly cross-sectional and limited to a single or few locations [22]. To address this gap in our knowledge, this study used annual data on locations of social service providers across the 48 continuous U.S. States from 1990 to 2014 and Census data for 1990 and 2010 to assess how spatial access to social services changed in high-poverty neighborhoods over this period. Also given that hospitals are also increasingly becoming stakeholders in local and regional decisions around urban design/planning, zoning and economic development planning, we also considered how spatial access to social services was linked to elements of urban form and built environments that might provide agglomorative advantages [6, 27–36].

Our analysis considers access to social services in relation to the presumed demand for social services, as measured by median household income and poverty rate. The goal of the analyses is to describe the history of the development and persistence of disparities in spatial access to social services and to place this history within the context of theories that explain why establishments locate within certain areas. This historical longitudinal analysis is necessary not only to critically assess the decisions made in past years and examine current issues and problematics, but to inform future decisions about where services should be located to assure access.

## Methods

### Identification of social service providers

We identified and compiled locations of social service providers across the continental, lower 48 U.S. states annually from 1990 to 2014 from the National Establishment Times Series (NETS). Since 1990, the NETS provides an annual, January census of U.S. business, nonprofit, institutional and government establishments that have a Data Universal Number System (DUNS) identifying number [37]. DUNS numbers are unique identification numbers issued to entities by Dun & Bradstreet (D&B), these numbers are issued and administered as part of D&B’s business of developing predictive credit ratings and scores for banking and insurance purposes [38]. The U.S. Federal Government requires all applicants for federal grants and cooperative agreements to have a DUNS number and requires DUNS numbers to be physical location specific [39, 40]. The Internal Revenue Service and the U.S. Small Business Administration state that each physical location of an entity should have their own unique DUNS number [39, 40]. DUNS numbers are required for the running businesses and not-for-profits, including establishing credit worthiness, setting contracts with suppliers, banking, applying for business certificates and getting business insurance [38]. Because State and Federal funded social service programs are commonly delivered through for-profit and not-for profit entities, and additionally because social service agencies commonly need business services, it is expected that the vast majority of social service agencies have DUNS numbers.

Through a partnership with D&B, Walls and Associates developed the longitudinal NETS product to capture dynamics of the U.S. economy [41, 42]. NETS is considered one of the most comprehensive establishment data sources available, effectively serving as a census of American businesses, organizations and institutions for each year since 1990 [42]. NETS contains over 300 variables per business for each year including company name, location (city, state, zip code, latitude and longitude), relocation history, eight digit modified Standard Industrial Classification (SIC) codes, sales volume, and number of employees. While NETS has primarily been used for research in economics to study job growth, and business formation, dissolution, and relocation [42–47], researchers have used NETS in health research to characterize neighborhood access to health-relevant businesses and institutions [41, 48–50].

All establishment addresses in NETS were geocoded to longitude and latitude coordinates using Esri ArcGIS software and a national street network reference data from NAVTEQ 2014. Addresses for each year from 1990 to 2014, were linked to 2010 US Census tract geometries. From NETS, we selected all establishments with SIC codes between 83,220,000 and 83,229,999, “Individual and Family Social Services Establishments”, which include 58 sub-classifications of service activity for entities “…primarily engaged in providing nonresidential social assistance to children and youth, the elderly, persons with disabilities, and all other individuals and families” [51]. The data include sole proprietorships, such as solo-practitioner clinicians who provides mental health services and large multi-location charities and not-for profits such as Catholic Charities. Counts of these establishments per 2010 Census tract geometries were calculated in each year from 1990 to 2014.

### Tract level compositional characteristics

The Longitudinal Tract Database (LTDB) was used to estimate population compositional characteristics of 2010 Census tract geometry areas in 1990 and 2010. The LTDB provides Census data harmonized from the 1990 to 2010 Decennial Censuses and aggregated to 2010 Census tract boundaries [52]. For these analyses, we used data on total population, count of people living below the federal poverty line, percent of population living below the poverty line (poverty rate), and inflation-adjusted median household income. Using data on the land area of 2010 Census tract geometries from the 2016 TIGER/Line files, we calculated population density of each tract. Density of social service providers was calculated both per tract area (land area in Km^2^) and as providers per 100 people living in poverty for each tract in 1990, 2000 and 2010.

### Other tract level built environment characteristics

As described previously, retail, commercial and institutional establishments that support the living needs of residents and people who work nearby, collectively referred to as “destinations that support living needs,” were identified using NETS [53]. We calculated the density of destinations that support living needs (per Km^2^ land area) for each Census tract in the U.S. for 1990. Also as described previously, we used data from The Center for Transit-Oriented Development to identify all rail transit stops in 2010 in the U.S., with internet research used to identify Census tracts with a rail transit stop in 1990 [53].

### Graphical and statistical analyses

Analyses were restricted to tracts that fell within a core-based statistical area (CBSA) in the continental U.S. and had a nonzero population in 1990. We stratified tracts into tertiles of population density and into quartiles of poverty rate using the respective distribution of both variables in CBSA in 1990. For tracts in the highest quartile of poverty rate we calculated the difference in inflation-adjusted median household income from 1990 to 2010 and classified tracts into quartiles of change in median household income. Separate quartile cut points for change in median household income were calculated for tracts in each tertile of population density.

We plotted graphs of mean annual density of social service providers per Km^2^ for tracts in each quartile of 1990 poverty rate, in each tertile of 1990 population density. Graphs were also plotted of mean annual count of social service providers per Km^2^ for tracts in the highest quartile of poverty rate in 1990 by quartile of change in inflation-adjusted median household income from 1990 to 2010. For tracts in the highest quartile of poverty rate in 1990, graphs were also plotted of count of social service providers per 100 persons living in poverty for 1990, 2000 and 2010, stratified by quartile of change in inflation-adjusted median household income from 1990 to 2010.

Generalized linear regression models, with a Gamma outcome distribution and identity link function, were fit with the density of social services providers per tract as the outcome variable; generalized estimating equations (GEE) were used to estimate model parameters. To avoid having zero values for the outcome variable, a violation of Gamma models, a value of 0.000005 was added to all observations, which is equal to one tenth of the smallest non-zero density of social services in the data set. Thus, the dependent variable is the count of social services agencies per Km^2^ within each tract, plus 0.000005, in each year of observation. The GEE accounted for correlation within Census tracts and used an autoregressive 1 structure to account for repeated observations within tracts across years. The regression models predicted annual tract-level social service agencies/Km^2^ using quartiles of 1990 poverty rate, with the lowest quartile of poverty rate set as the referent group, year of observation, and year*quartile of 1990 poverty rate as independent variables. We performed separate analyses for tracts falling in the 1^st^, 2^nd^ and 3^rd^ tertiles of population density. To assess the effect of quartiles of change in median household income from 1990 to 2010 on the density of social services in tracts in the highest quartile of poverty in 1990, we implemented GEE models with count of social services agencies per Km^2^ within each tract plus 0.000005 in each year of observation as the dependent variable and used variables for year of observation, quartile of change in median household income and year*quartile of change in median household income as independent variables.

We also conducted cross-sectional analyses of associations between quartile of change in median household income from 1990 to 2020 in high-poverty tracts and 1990 median household income and 1990 built environment features. We analyzed associations between quartile of change in median household income from 1990 to 2020 (predictor) and social services per Km^2^ (dependent variable) and between quartile of change in median household income from 1990 to 2020 (predictor) and destinations that support living needs per Km^2^ (dependent variable) using a generalized linear model with a gamma distribution. We cross-sectionally analyzed the association between quartile of change in median household income from 1990 to 2020 (predictor) and 1990 median household income (dependent variable) using linear regression. We also cross-sectionally analyzed the association between quartile of change in median household income from 1990 to 2020 (predictor) and the presence of rail transit stops in 1990, coded and present vs absent as the dependent variable, using logistic regression.

## Results

In total, there were 66,737 Census tracts located within CBSAs with nonzero populations in 1990. Tracts in the lowest tertile of population density had a combined estimated 1990 population of 66,716,779 (7,610,576 living in poverty), tracts in the 2^nd^ tertile of population density had a combined 1990 population of 78,062,889 (7,716,125 living in poverty) and tracts in the highest tertile of population density had a combined 1990 population of 85,192,826 (13,086,511 living in poverty).

**Fig. 1:**
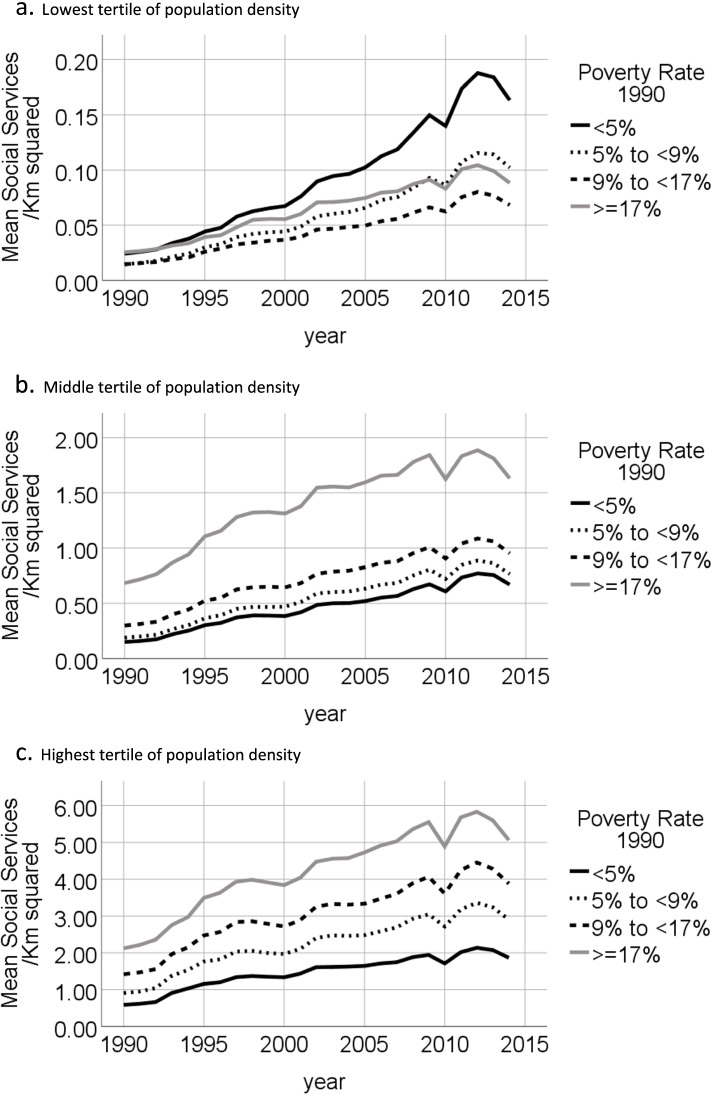
Annual number of social service agencies per Km^2^ in 2010 tract areas by quartiles of poverty rate measured in 1990 for tracts located within U.S. core-based statistical areas

For tracts in the lowest tertile of population density the mean number of social services providers per Km^2^ was higher in tracts with the lowest poverty rates in 1990, while for tracts in the middle and highest tertiles of population density, the mean number of social services providers per Km^2^ was higher in tracts with higher poverty rates in 1990 (see Fig. 1). GEE analyses of tracts in the lowest tertile of population density did not converge. GEE analyses of tracts in the middle and highest tertile of population density found that in 1990, compared to tracts in the lowest quartile of poverty, the tracts in 2^nd^, 3^rd^ and 4^th^ (highest) quartiles of poverty had significantly higher densities of social services providers (see Table 1). Additionally, compared to tracts in the lowest quartile of poverty, the estimated linear increase in the density of social service providers by year was significantly higher for tracts in the 2^nd^, 3^rd^ and 4^th^ (highest) quartiles of poverty compared to the first quartile (see Table 1).

**Table 1:** Differences in the tract level density of social service providers by quartiles of 1990 poverty rate and 1990 poverty rate by year interactions

Predictor Variables	Difference in provider counts/Km^2^ (95% CI) *P*-value For tracts in the second tertile of population density (248—1,479 people/Km^2^)	Difference in provider counts/Km^2^ (95% CI) *P*-value For tracts in the third tertile of population density (> 1,479 people/Km^2^)
Quartile of 1990 poverty rate		
1^st^ Quartile (low poverty)	Ref	Ref
2^st^ Quartile	0.04 (0.02, 0.05) *p* < 0.001	0.30 (0.20, 0.41) *p* < 0.001
3^st^ Quartile	0.14 (0.10, 0.17) *p* < 0.001	0.79 (0.66, 0.93) *p* < 0.001
4^st^ Quartile (high poverty)	0.48 (0.43, 0.54) *p* < 0.001	1.45 (1.33, 1.57) *p* < 0.001
Quartile of 1990 poverty rate * Year		
1^st^ Quartile * Year	Ref	Ref
2^st^ Quartile * Year	0.003 (0.002, 0.005) *p* < 0.001	0.04 (0.02, 0.05) *p* < 0.001
3^st^ Quartile * Year	0.009 (0.005, 0.013) *p* < 0.001	0.06 (0.05, 0.07) *p* < 0.001
4^st^ Quartile* Year	0.03 (0.025, 0.033) *p* < 0.001	0.09 (0.08, 0.1) *p* < 0.001

Tracts in the highest quartile of poverty in 1990 were grouped into quartiles of change in inflation-adjusted median household from 1990 to 2010. Among these high-poverty tracts, some tracts experienced increases in household income from 1990 to 2010 (all tracts in the fourth quartile of change in median household income) and some experienced declines in household income (all tracts in the first quartile of change in median household income – see Table 2). For these high-poverty tracts in the middle and highest tertiles of population density, tracts that experienced the largest 1990 to 2010 gain in median household began the observation period (1990) with the highest density of destinations that support living needs, the highest density of social services, and were most likely to have rail transit stops (see Table 2). However, tracts that experienced the largest gains in median household income between 1990 and 2010 were not the tracts with the lowest income in 1990.

**Table 2:** Characteristics of tracts in the highest quartile of 1990 poverty rate by change in inflation adjusted median household income from 1990 to 2010

Quartiles of Change in Inflation-Adjusted Median Household Income 1990–2020	Median Household Income, 1990 Mean	Density^a^ of Destinations that Support Living Needs, 1990 Mean, Median	Percent of Tracts with Rail Transit Stops, 1990	Density^b^ of Social Service Providers, 1990 Mean, Median
Lowest Tertile of Population Density (< 248 people/Km^2^)				
2003Q1 < -$1,390	$21,401*	2*, 1	1%	0.03, 0
Q2 -$1,390 to < $4,195	$19,407*	1*, 0	0%	0.02*, 0
Q3 $4,195 to < $9,917	$19,223*	1*, 0	0%	0.02*, 0
Q4 > = $9,917	$20,103	4, 0	1%	0.03, 0
Middle Tertile of Population Density (248–1,479 people/Km^2^)				
Q1 < -$5,808	$21,689*	17*, 10	1%*	0.39*, 0
Q2 -$5,808 to < -$934	$18,001	20*, 12	2%*	0.57*, 0
Q3 -$934 to < $4,472	$16,483*	23*, 13	1%*	0.80, 0
Q4 > = $4,472	$17,513	34, 13	4%	0.98, 0
Highest Tertile of Population Density (> 1,479people/Km^2^)				
Q1 < -$7,184	$22,600*	61*, 36	7%*	1.51*, 0
Q2 -$7,184 to < -$1,765	$18,530*	64*, 36	6%*	1.77*, 0
Q3 -$1,765 to < $4,142	$16,480*	68*, 37	6%*	2.07*, 1
Q4 > = $4,142	$17,792	101, 49	12%	3.14, 1

^a^Count of destinations per Km^2^

^b^Count of Social service providers per Km^2^

^*^P < 0.05 for difference compared to the 4^th^ quartile (largest) of change in inflation-adjusted median household income from 1990–2020

**Fig. 2:**
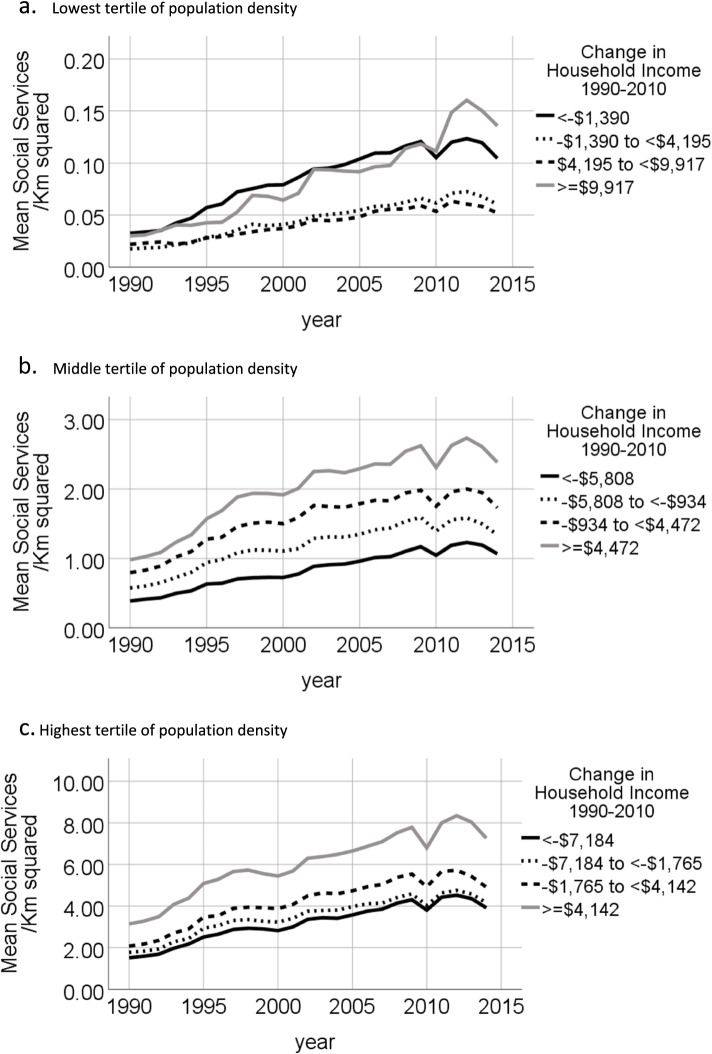
Number of social service agencies per Km^2^ in high poverty tracts by quartiles of change in inflation adjusted median household income from 1990 to 2010. High poverty tracts are defined as 2010 tracts in the highest quartile of poverty rate in 1990, among tracts located within U.S. core-based statistical areas

Panels A-C of Fig. 2 show the density of social service providers for tracts in the highest quartile of poverty in 1990 by year and by quartile of change in household income from 1990 to 2010. For tracts in the middle and highest tertile of population density in 1990 and that were also in the highest quartile of poverty rate in 1990, the mean number of social service providers per Km^2^ increased through time but the extent of the increase was related to changes in median household income through time. High poverty tracts that experienced the largest increases in median household income from 1990 to 2010 had the highest density of social services providers in 1990 and saw the largest increases in the density of social service providers from 1990 to 2014. High poverty tracts that experienced the largest declines in the household income from 1990 to 2010 had the lowest density of social services in 1990 through to 2014. GEE analyses of tracts in the 1^st^ tertile of population density did not converge. GEE analyses of tracts in the middle and highest tertiles of population density found that in 1990, the tracts in 2^nd^, 3^rd^ and 4^th^ quartiles of change in household income had significantly higher densities of social services providers than tracts in the 1^st^ quartile (see Table 3). Additionally, the linear increase in the density of social service providers by year was significantly higher for tracts in the 2^nd^, 3^rd^ and 4^th^ quartiles of change in household income than for tracts in the 1^st^ quartile (see Table 3).

**Table 3:** Differences in the tract level density of social service providers by quartiles of change in 1990 to 2010 median household income and year for tracts that were in the highest quartile poverty in 1990

Predictor Variables	Difference in provider counts/Km^2^ (95% CI) *P*-value For tracts in the second tertile of population density (248—1,479 people/Km^2^)	Difference in provider counts/Km^2^ (95% CI) *P*-value For tracts in the third tertile of population density (> 1,479people/Km^2^)
Quartiles of Change in Inflation-Adjusted Median Household Income 1990–2010		
1^st^ Quartile	Ref	Ref
2^nd^ Quartile	0.15 (0.04, 0.26) *p* = 0.006	0.24 (0.03, 0.44) *p* = 0.024
3^rd^ Quartile	0.35 (0.21, 0.49) *p* < 0.001	0.50 (0.28, 0.73) *p* < 0.001
4^th^ Quartile	0.53 (0.37, 0.7) *p* < 0.001	1.53 (1.23, 1.83) *p* < 0.001
Quartiles of Change in Inflation-Adjusted Median Household Income 1990–2010 * Year		
1^st^ Quartile * Year	Ref	Ref
2^st^ Quartile * Year	0.01 (0.004, 0.02) *p* = 0.001	0.004 (-0.01, 0.02) *p* = 0.583
3^st^ Quartile * Year	0.02 (0.01, 0.03) *p* = 0.001	0.03 (0.01, 0.05) *p* = 0.001
4^st^ Quartile * Year	0.04 (0.03, 0.06) *p* < 0.001	0.09 (0.07, 0.11) *p* < 0.001

For tracts in the second tertile of population density the quartiles of change in median household income were: Q1 < -$5,805; Q2 -$5,808 to < -$934; Q3 -$934 to < $4,472; and Q4 >  = $4,472.

For tracts in the third tertile of population density the quartiles of change in median household income were: Q1 < -$7,184; Q2 -$7,184 to < -$1,765; Q3 -$1,765 to < $4,142; and Q4 >  = $4,142.

**Fig. 3:**
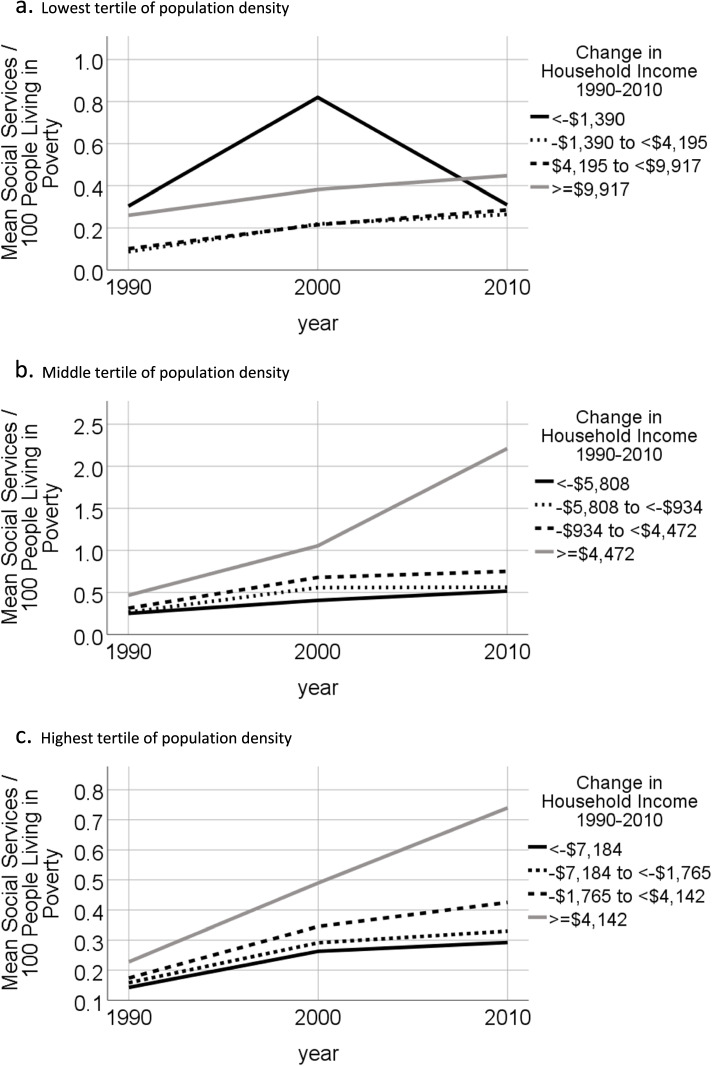
Number of social services agencies per 100 people living in poverty in high poverty tracts by quartiles of change in inflation adjusted median household income from 1990 to 2010. High poverty tracts are defined as 2010 tracts in the highest quartile of poverty rate in 1990, among tracts located within U.S. core-based statistical areas

Figure 3 shows the number of social service providers per 100 people living in poverty in 1990, 2000 and 2010 for tracts in the highest quartile of poverty in 1990, by quartile of change in household income from 1990 to 2010. The trends observed over time for social services per 100 people living in poverty and social service per land area (Fig. 2) were similar. For tracts in the middle and highest tertiles of population density, access to social services per 100 people living in poverty in 1990 was highest in tracts that experienced the largest gains in household income between 1990 and 2010, and access rose most steeply in these tracts from 1990 to 2010.

## Discussion

In high-poverty, higher population density Census tracts, a spatial mismatch emerged during the period 1990 to 2010 between the availability of social services and the expected need for social services as the population characteristics of neighborhoods changed. High poverty tracts that experienced declines in median household income from 1990 to 2010, began the period with the lowest spatial access to social services and saw the smallest increases in access to social services. This was true when access was measured as services per Km^2^ area of the tract or when measured as services per 100 people living in poverty in the tract. We also observed that in 1990, in high poverty tracts, the density of social service agencies was highest in tracts that experienced the highest gains in median household income from 1990 to 2010. These were also the tracts that in 1990 had more amenities that “support living needs,” such as public transit, which facilitates easier commute for both workers and clients, and a more robust retail and commercial environment with shops, restaurants, and banks as well as vendors and suppliers that support the running of the providers. These findings have implications for hospital’s success in addressing patient’s unmet social needs and suggest that hospital’s increasing role as a stakeholder in local urban planning and economic development planning could be used to expand spatial access to social services [6, 27–36].

### Clustering of services to maximize resources

Researchers examining the nonprofit sector note that such organizations tend to cluster [25] because of “cost reducing agglomeration effects” [14]. Agglomeration economics posits that co-location increases the availability of resources necessary for an organization and provides other advantages, such as allowing for specialization of the services an organization provides, better matching between employers and workers, and diffusion of innovation through tighter professional and social networks [54]. For social service providers, spatial clustering of services may also allow for the more efficient provision of specialized services to families with multiple needs. Studies of Los Angeles County investigating agglomeration phenomena for services to families with children and to the homeless, provide some evidence that rather than being solely determined by proximity to potential users, social service location decisions are influenced by functional linkages with similar providers and a willingness of communities to host service facilities [55, 56]. They found that children’s service organizations in LA County were concentrated in middleclass areas, where other related services were located. Volunteers and donations were also more abundant than in poorer areas, and the political culture was more open to controversial human service facilities than in White-dominant suburban communities prone to “not in my backyard” (NIMBY)ism [57].

Our analyses suggest that in 1990, among high-poverty tracts, social service providers were clustered in tracts with amenities that “support living needs,” such as public transit, which facilitates easier commute for both workers and clients, and a more robust retail and commercial environment with shops, restaurants, and banks as well as vendors and suppliers that support the running of the providers. This co-location of providers and retail may, in part, be driven by zoning regulations that commonly establish some areas as being solely for residential uses. Unsurprisingly, our analyses also indicate that tracts that experienced increases in median household income from 1990 to 2010, were richer in amenities that support living needs in 1990.

### Marketization of social services

Contrary to an expectation that the availability of social services would expand in high-poverty tracts that experienced further economic decline, providers per Km^2^ and access per 100 individuals living in poverty increased most among high-poverty tracts that experienced increases in household income through time. These seemingly unexpected trends can be explained by the observation that welfare has evolved to become increasingly marketized through time, in a way that would favor provision of services in high-poverty tracts that experienced income growth. The “Marketization of Welfare,” describes the shifting balance of welfare services towards those who can pay, consequently decreasing access for the disadvantaged [26]. The delivery of social welfare programs in the U.S. involves extensive and complex partnership between government and private nonprofit and for-profit providers, with private institutions funded through government contracts handling the delivery of services. During the 1970 and 1980s, the proportion of income that non-profit social welfare providers generated through fees and commercial income increased and there was a concurrent rise in the for-profit sector in delivering social welfare programs [26]. Subsequent research through the early 2000s showed continued growth of commercial revenue among human services nonprofits [58]. Thus, welfare agency budgets often rely on a combination of government funds or fees and commercial income from paying clients, or reimbursement from client’s insurance providers or employee assistance programs. Such financial models suggest an explanation for the trends in spatial access observed in the data; high-poverty tracts on an upward economic trajectory provided an attractive mix of residents, those in need of government funded services and increasingly those with the means or insurance coverage to utilize fee-for-service products. Conversely, high-poverty tracts on downward economic trajectories were expected to be the least attractive areas for providers with the prevailing budget models. The apparent preference for social service providers to locate in high-poverty tracts that were on an upward economic trajectory may reflect a form of “client creaming” by fee-motivated providers [59].

### Strengths and limitations

Our big data approach to tracking changes in spatial access to social services has several strengths including multiple decades of data, a national scope, a uniform approach to identifying social service providers, and the incorporation of both Census and urban form data. However, this approach lacks the nuance of other studies that focused in depth on single cities, specific types of services, had administrative, organizational and/or funding data for providers or that could focus on agency service areas. The number of social services outlets in a particular location is not necessarily indicative of the level of services available in that location due to differential funding, both governmental and private, available to different providers of varying sizes and capacity [16]. Our data did not capture the size of the organizations or the funding they commanded. While the Federal Government directs entities to have a separate DUNS number for each physical location, it is possible that some agencies’ satellite locations did not have a unique DUNS number and the NETS undercounts social service locations. The use of spatial units such as Census units (Blocks Groups and Tracts), ZIP Codes and counties as a unit of analysis is common in urban health and spatial access research, for instance see [13, 18, 60–62]. However, the use of such spatial units can cause boundary issues in analyses and interpretation [62]. It is clearly possible that residents of a tract with no or relatively few services could access services in a neighboring or nearby tract. However, tract level analyses do allow for the linking of Census and other spatial data to the NETS data and provide a national picture of how the location of social services has evolved as population characteristics have changed over a 25-year period.

## Conclusion

Overall, spatial access to social service providers was higher in high-poverty as compared to low-poverty tracts in 1990 and access increased from 1990 to 2014. However, high-poverty tracts that experienced increases in median household income from 1990 to 2010 had higher spatial access to social services in 1990 and had the highest rate of increase in services through 2014. Conversely, high-poverty tracts that experienced further economic decline from 1990 to 2010 had the lowest spatial access to services in 1990 and realized the lowest rate of increase in services from 1990 to 2014. Agglomeration benefits and the marketization of welfare may explain the mismatch between spatial access to services and populations presumed to be in need.

These historical trends have implications for the success of hospital’s current strategies to meet the social and economic needs of patients and improve health outcomes [1, 3, 6–11]. Agglomeration benefits predict that social services will spatially cluster and the analyzes presented here suggest that clustering is linked to other elements of urban built form, such as a more robust retail, commercial and institutional environment and access to rail transit. As hospitals and health care systems are increasingly becoming stakeholders in local urban planning, zoning and economic development decisions, they should consider how decisions about urban form may influence spatial access to social services. Hospital system’s advocacy for transit-oriented design and mixed-land use may create the conditions that attract social service agencies into hospital catchment areas [35]. Given the significant role that market forces play in determining the placement of services, attention to interdisciplinary theories across urban planning, economics and social and health services research is needed to improve spatial access to social services.

## Data Availability

Data cannot be shared publicly due to the terms and conditions of the licensed data. Data are available for researchers who meet the criteria to work with the licensed data. Contact gslovasiresearch@gmail.com or visit https://sites.google.com/view/recvd-team-project-site/home for more information. All other questions contact Gina S Lovasi PhD, MPH at gsl45@drexel.edu.
